# Supplementary material for: Thickness determines microbial community structure and function in nitrifying biofilms via deterministic assembly
Source: Sci Rep. 2019 Mar 25;9:5110. doi: 10.1038/s41598-019-41542-1 (PMC6434030; doi:10.1038/s41598-019-41542-1)
Supplement: Supplementary file 1 — Supplementary Information [file 41598_2019_41542_MOESM1_ESM.pdf]

Supplementary material for:

Thickness determines microbial community structure and function in nitrifying biofilms via deterministic assembly.

Carolina Suarez, Maria Piculell, Oskar Modin, Silke Langenheder, Frank Persson, Malte Hermansson

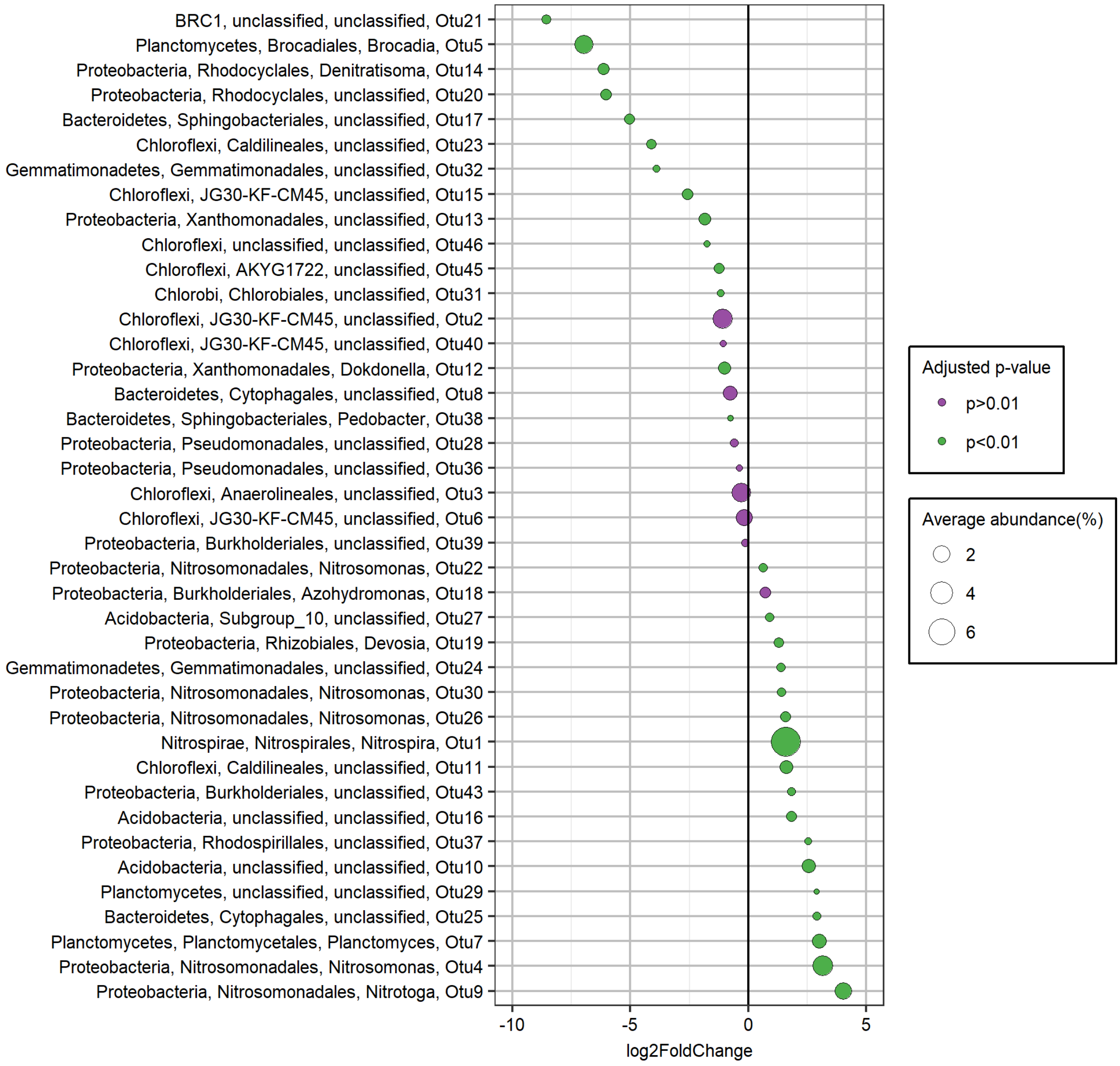

Fig S1: Log2fold (DESeq2) changes for the 40 most abundant SVs based on average abundance**.** Phylum, order and genus classification are shown. Each circle represents an SV. The size of the circle is proportional to the total sequence read abundance for the SV. A negative log2 fold change indicates that SV are more abundant in Z400 biofilm, while a positive log2 fold change indicates SVs more abundant in Z50 biofilms.

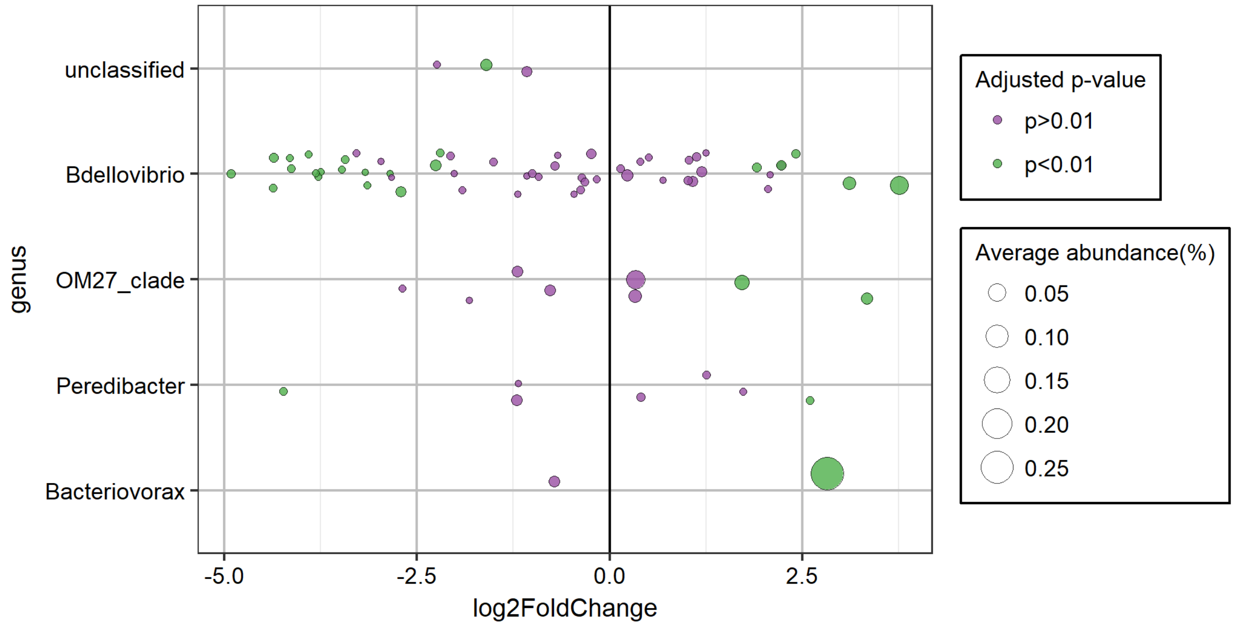

FIG S2: Log2fold (DESeq2) changes for *Bdellovibrionales* SVs. Genus classification is shown. Each circle represents an SV. The size of the circle is proportional to the total sequence read abundance for the SV. A negative log2 fold change indicates that SV are more abundant in Z400 biofilm, while a positive log2 fold change indicates SVs more abundant in Z50 biofilms. SVs with a NA $p_{(adj)}$ value (DESeq2) are not shown.

Supplemental Material and Methods

Fluorescence in situ hybridization

Carriers were removed directly from the pilot reactor and fixed in 4% paraformaldehyde for 8h at 4°C. Following fixation the biofilms were rinsed by immersing the carriers twice in phosphate-buffered saline (PBS) for 15 min, after which the carriers were stored in 50:50 PBS-ethanol at -20°C until analysis.

Preparation of qFISH:

Biofilm suspensions were used for qFISH by brushing off the fixed biofilm from three Z50 and three Z400 carriers and homogenizing the biomass in PBS. The biofilm suspensions were stored in 50:50 PBS-ethanol at -20˚C until use. Prior to FISH, 15 µl aliquots were spotted onto SuperFrost Plus Gold microscope slides (Menzel GmbH, Braunschweig, Germany). A hydrophobic barrier frame was applied to the glass slides around the regions containing the biofilm suspensions by using a Liquid Blocker Mini Pap Pen (Thermo Fisher Scientific, USA)

Differences between qFISH and sequence abundance

qFISH and Miseq are complementary methods and differences are expected because methods are based on different principles; sequencing detect rDNA and FISH detect rRNA. For instance, we noticed that the signal strength of the AMX820 probe was low for many anammox cells, which can lead to difficulties in distinguish signal from background during image segmentation. Also differences in ribosomal gene copy number, DNA extraction methods as well as e-DNA could influence sequencing results [1,2]. Also, underestimation of *Nitrosomonas* in 16S rRNA PCR methods compare to qFISH has been noticed in several studies [3,4], perhaps depending on relatively high ribosomal content even in inactive cells [5].

Hence, all methods suffer from limitations and multiple methods provide important complementary information.

Preparation of cryosections:

After fixation, a 1 cm^2 section of the carrier, was selected and cut for cryosectioning. To remove the biofilm from the plastic, the carrier section was placed in a container filled with Optimal Cutting Temperature (O.C.T.) compound (VWR, USA) and stored overnight at 4˚C. The next day the container was placed in a container with dry ice until the O.C.T. compound was completely frozen, after which the plastic carrier section could be removed while biofilm remained attached to the compound. The intact biofilm was covered with more compound before it was re-frozen and stored at -80˚C until use. Biofilm cryosections were obtained using a HM550 microtome cryostat (MICROM International GmbH, Germany) at -20˚C, cutting out 20–25 µm thick cross-sections of the biofilm which were collected on SuperFrost Plus Gold microscope slides (Menzel GmbH, Braunschweig, Germany). Finally, the slides were covered with a low melting agarose layer to avoid biomass detachment during FISH. FISH was performed at 46˚C for 2 h [6]. When probes with different hybridization stringency optima were applied to the same sample, consecutive hybridizations were performed, beginning with the probe(s) requiring the most stringent conditions [6]. Competitor probes were added as unlabeled oligonucleotides in equimolar amounts as the labeled probes to the hybridization mix, in order to increase hybridization specificity. For qFISH a permeable nucleic acids stain, SYTO 40 was used as reference (Thermo Fisher Scientific, USA) and specific population probes (see Supplementary Table S1) were labeled with Cy3 or Cy5. During FISH on cryosections populations were labelled with FAM, Cy3, Cy5 and one of the probes was double labeled with Cy3 and Cy5 [7]. Labeled oligonucleotides were synthesized by Eurofins Genomics (Germany). Counterstaining with SYTO 40 (Thermo Fisher Scientific,

USA) was done at 10 µM for 30 minutes. After FISH and counterstaining, the slides were mounted in the antifadant Prolong Diamong Antifade (Thermo Fisher Scientific, USA).

Table S1. FISH probes used in this study

Probe	Target	Formamide	Reference
Cla6192	*Nitrosomonas* cluster 6	35%	[8]
NEU	*Nitrosomonas halophila/eutropha/europaea*	40%	[9]
Ntspa662	*Nitrospira*	35%	[10]
Ntoga122	*Nitrotoga*	40%	[11]
AMX820	*Brocadia* and *Kuenenia*	40%	[12]

Microscopy and image analysis

Images were acquired using a Zeiss LSM700 confocal microscope (Carl Zeiss, Germany), using laser lines of 405, 488, 555 and 639 nm at settings of frame mode and averaging = 4. The same pinhole size was used in all channels, equivalent to 1AU for the Cy5 channel. Images were obtained with a 40×/1.3 plan-apochromat oil objective. To create composite images of large size, the tile function of the Zeiss ZEN2012 software was used. Contrast of SYTO 40 was lowered in images of cryosections to facilitate visualization of populations; intensity of the Cy5 channel was increased due to low Cy5 signal for the double labelled probe. For qFISH pictures were taken from 30 random fields of view for each target populations in each carrier type.

The relative abundances of the target populations for qFISH was estimated on biofilm suspensions as the ratios of the FISH-targeted biovolumes of the specific populations to the total FISH-targeted biovolumes (SYTO 40, Table S1) in daime2.1 [13]. After importing the image channels, noise reduction (4 voxels) and median filtering was used (1 voxel). For all channels, low intensity pixels, below a threshold of 75, were removed. For 2-D segmentation,

biomass detection was done by thresholding using the RATS-L algorithm. Boolean operations were used in the image masks to remove signal no present in the reference channel. Biovolume fraction was calculated using the SYTO-channel as reference.

Targets, hybridization conditions and references for the FISH probes are described in table S1.

Biofilm structure staining

The biofilm matrix was stained in cryosections with FilmTracer SYPRO Ruby biofilm matrix stain (Thermo Fisher Scientific, USA) using 200µl for 30 min. The slides were then mounted with Prolong Diamong Antifade. For microscopy a laser line of 488nm was used, with settings of frame mode and averaging = 4. A pinhole size was used equivalent to 1AU. Images were obtained with a 40×/1.3 plan-apochromat oil objective.

DNA extraction and 16s sequencing

Biomass was removed from the Z-carriers by brushing it into 4 ml of sterile water, with the resulting suspension being transferred to a 15ml centrifuge tube. The suspension was centrifuged at 4653g for 3 minutes and the supernatant was discarded. 978 µl of sodium phosphate buffer and 122 µl of MT buffer, of the FastDNA SPIN kit for soil (MP Biomedicals), were added to the 15 ml centrifuge tubes. The biofilms were resuspended by pipetting and 1.1 ml of the suspensions were transferred to Lysing Matrix E tubes. FastPrep homogenization and subsequent purification steps were done according to manufacturer instructions.

PCR amplification of the v4 region of the 16S rRNA gene was done with primers 515F' [14] and 806R [15], using dual indexing of the primers [16]. 40 ng of template were amplified using a Phusion Hot Start II DNA Polymerase (Thermo Fisher Scientific, USA). The following PCR program was used: activation (98°C, 30 s); 30 cycles of denaturation (98°C, 10 s), annealing

(56°C, 30 s) and elongation (72°C, 15 s); followed by final elongation (72°C, 10 min). PCR products were purified with the MagJET NGS Cleanup and Size Selection Kit (Thermo Fisher Scientific, USA). DNA concentrations of the purified products were measured using a Qubit 3.0 fluorometer (Thermo Fisher Scientific, USA), using the dsDNA HS assay kit (Thermo Fisher Scientific, USA). The obtained products were quality checked by standard gel electrophoresis. Purified PCR products were pooled in equimolar amounts. Quality control of the pooled PCR product was performed on a TapeStation 2200 (Agilent Technologies). PhiX control library was spiked in at 7.5%. Sequencing was performed on an Illumina MiSeq using the MiSeq Reagent Kit v2.

Raw sequence reads were processed in Usearch (version 10). Paired-end reads were merged with the fastq_mergepairs command allowing a maximum of 12 mismatches in the alignment. This resulted in 2 113 324 merged reads. The merged reads were quality filtered using a maximum expected error cutoff of 0.5 and a minimum sequence length of 200 bp. The quality filtered reads were used as input to the Unoise algorithm [17] to generate sequence variants. A minimum abundance threshold of 4 was specified in the unoise3 command. This means that sequence variants were discarded if they were represented by fewer than 4 quality filtered reads across all samples. In total, 1 657 741 reads were mapped to 3692 sequence variants. Taxonomic classification was done with the sintax algorithm [18] and the SILVA 128 training set database was used for taxonomic classification [19].

MATHEMATICAL MODELING

Detailed model description

The goal of the mathematical model was to simulate dissolved oxygen (DO) concentration profiles and ammonium oxidation rates in biofilms on the Z400 and Z50 carriers. The notation used in the model is shown in Supplementary Table S2.

Table S2. Notation used in the DO model.

Parameter	Definition	Units
Biomass and substrate components		
X_H	Concentration of heterotrophic bacteria	$gCOD\ m^{-3}$
X_A	Concentration of ammonium-oxidizing bacteria (AOB)	$gCOD\ m^{-3}$
X_N	Concentration of nitrite-oxidizing bacteria (NOB)	$gCOD\ m^{-3}$
X_I	Concentration of inert solids	$gTS\ m^{-3}$
S_O	Concentration of dissolved oxygen	$gO_2\ m^{-3}$
S_N	Concentration of nitrite	$gN\ m^{-3}$
S_A	Concentration of ammonium	$gN\ m^{-3}$
S_C	Concentration of soluble, readily biodegradable organics	$gCOD\ m^{-3}$
Kinetic and stoichiometric coefficients		
μ_{Hmax}	Maximum growth rate of heterotrophs	d^{-1}
b_H	Aerobic endogenous respiration rate constant for heterotrophs	d^{-1}
K_{OH}	Affinity constant for oxygen of heterotrophs	$gO_2\ m^{-3}$
K_C	Affinity constant for organics of heterotrophs	$gCOD\ m^{-3}$
Y_H	Yield coefficient for heterotrophs growing aerobically	$gCOD_{XH}\ g^{-1}COD_{SC}$
μ_{Amax}	Maximum growth rate of AOB	d^{-1}
b_A	Aerobic endogenous respiration rate constant for AOB	d^{-1}
K_{OA}	Affinity constant for oxygen of AOB	$gO_2\ m^{-3}$
K_A	Affinity constant for ammonium of AOB	$gN\ m^{-3}$
Y_A	Yield coefficient for AOB	$gCOD_{XA}\ g^{-1}NH_4\text{-}N$
μ_{Nmax}	Maximum growth rate of NOB	d^{-1}
b_N	Aerobic endogenous respiration rate constant for NOB	d^{-1}
K_{ON}	Affinity constant for oxygen of NOB	$gO_2\ m^{-3}$
K_N	Affinity constant for NO_2^- of NOB	$gN\ m^{-3}$
Y_N	Yield coefficient for NOB	$gCOD_{XN}\ g^{-1}NO_2\text{-}N$
f_{XI}	Fraction live biomass being convert to inert material during endogenous respiration	
i_{NX}	Nitrogen content in biomass	$gNH_4\text{-}N\ g^{-1}COD$

Physical parameters		
D_{W_O}	Diffusion coefficient of oxygen in water	$m^2\ s^{-1}$
D_{W_N}	Diffusion coefficient of nitrite in water	$m^2\ s^{-1}$
D_{W_A}	Diffusion coefficient of ammonium in water	$m^2\ s^{-1}$
D_{W_C}	Diffusion coefficient of organic carbon in water	$m^2\ s^{-1}$
D_e	Effective diffusion coefficient, i.e. diffusion coefficient in biofilm	$m^2\ s^{-1}$
f_{VS}	Fraction of the total solids that is live, active bacteria	
fX_H	Fraction of the live bacteria that is aerobic heterotrophs	
fX_A	Fraction of the live bacteria that is AOB	
fX_N	Fraction of the live bacteria that is NOB	
$S_{O,bulk}$	Concentration dissolved oxygen in bulk liquid	$gO_2\ m^{-3}$
$S_{N,bulk}$	Concentration nitrite in bulk liuid	$gN\ m^{-3}$
$S_{A,bulk}$	Concentration ammonium in bulk liquid	$gN\ m^{-3}$
$S_{C,bulk}$	Concentration organic carbon in bulk liquid	$gCOD\ m^{-3}$
L	Biofilm thickness	m
Δx	Thickness of layer in biofilm	m
X_{TS}	Biofilm density	$gTS\ m^{-3}$
δ_{BL}	Bulk liquid-biofilm diffusion boundary layer thickness	M

Components

The model included three biomass components: aerobic heterotrophs (X_H), AOB (X_A), and NOB (X_N). It calculated the diffusion and conversions of four soluble components: DO (S_O), nitrite (S_N), ammonium (S_A), and biodegradable organic carbon (S_C).

Biochemical conversions

The activities of the three microbial groups (X_H, X_A, and X_N) were described using Monod kinetics. In total six kinetic equations described the rates of growth and decay (Supplementary Table S3). Aerobic oxidations of S_C, S_A, and S_N were considered. Denitrification and anammox were not included in the model because those processes were assumed to have only minor effect on the DO concentration profiles. A stoichiometric matrix (Supplementary Table S4) linked the kinetic equations to conversion rates of the soluble components. The conversion rate for a component of interest can be calculated using Equation 1. The kinetic

and stoichiometric coefficient values used as default input to the model are shown in Table S5.

$$r_i = \sum_{j=1}^{j=9} x_{ij} \cdot p_j \qquad (\mathbf{1})$$

where r_i is the conversion rate of component i, x_{ij} is the stoichiometric coefficient for component i and process j, and p_j is the rate of process j.

Table S3. Kinetic rate expressions used in the DO model.

#	Process	Equation
	Heterotrophs	
0	Aerobic growth	$\mu_{Hmax} \cdot \frac{S_O}{K_{OH} + S_O} \cdot \frac{S_C}{K_C + S_C} \cdot X_H$
1	Aerobic endogenous respiration	$b_H \cdot \frac{S_O}{K_{OH} + S_O} \cdot X_H$
	AOB	
2	Aerobic growth	$\mu_{Amax} \cdot \frac{S_O}{K_{O\_A} + S_O} \cdot \frac{S_A}{K_A + S_A} \cdot X_A$
3	Aerobic endogenous respiration	$b_A \cdot \frac{S_O}{K_{OA} + S_O} \cdot X_A$
	NOB	
4	Aerobic growth on NO_2^-	$\mu_{Nmax} \cdot \frac{S_O}{K_{ON} + S_O} \cdot \frac{S_N}{K_N + S_N} \cdot X_N$
5	Aerobic endogenous respiration	$b_N \cdot \frac{S_O}{K_{ON} + S_O} \cdot X_N$

Table S4. Stoichiometric matrix used in the DO model

**Comp. (*i*)**	**S0**	**S1**	**S2**	**S3**	
**Process (*j*)**	S_O (gO_2 m^{-3})	S_{NO2} (gN m^{-3})	S_{NH} (gN m^{-3})	S_C (gCOD m^{-3})	Units of rates
**P0**	$1\text{-}1/Y_H$		$-i_{NX}$	$-1/Y_H$	$gCOD_{XH}$ m^{-3} d^{-1}
**P1**	$f_{XI}\text{-}1$		$i_{NX}*(1\text{-}f_{XI})$		$gCOD_{XH}$ m^{-3} d^{-1}
**P2**	$3.43*(i_{NX} -1/Y_A)$	$-i_{NX} +1/Y_A$	$-1/Y_A$		$gCOD_{XA}$ m^{-3} d^{-1}
**P3**	$f_{XI}\text{-}1$		$i_{NX}*(1\text{-}f_{XI})$		$gCOD_{XA}$ m^{-3} d^{-1}

**P4**	$-1.14/Y_N$	$-1/Y_N$	$-i_{NX}$		$gCOD_{XN}\ m^{-3}\ d^{-1}$
**P5**	$f_{XI}-1$		$i_{NX}*(1-f_{XI})$		$gCOD_{XN}\ m^{-3}\ d^{-1}$

Table S5. Kinetic and stoichiometric coefficients used in the DO model.

**Coefficient**	**Value**	**Reference**
Heterotrophs		
μ_{Hmax}	$4\ d^{-1}$	[20]
b_H	$0.2\ d^{-1}$	[21]
K_{OH}	$0.2\ gO_2\ m^{-3}$	[21]
K_C	$5\ gCOD_{SC}\ m^{-3}$	[20]
Y_H	$0.67\ gCOD_{XH}\ g^{-1}COD_{SC}$	[20]
AOB		
μ_{Amax}	$1.4\ d^{-1}$	[22]
b_A	$0.3\ d^{-1}$	[22]
K_{OA}	$0.3\ gO_2\ m^{-3}$	[23]
K_A	$2.4\ gN\ m^{-3}$	[23]
Y_A	$0.15\ gCOD_{XA}\ g^{-1}N$	[23]
NOB		
μ_{Nmax}	$1.1\ d^{-1}$	[23]
b_N	$0.2\ d^{-1}$	[22]
K_{ON}	$0.1\ gO_2\ m^{-3}$	[23]
K_N	$0.238\ gN\ m^{-3}$	[23]
Y_N	$0.041\ gCOD_{XN}\ g^{-1}N$	[23]
**Other**		
f_{XI}	$0.2\ gCOD_{XI}\ g^{-1}COD_{XH,XA,XN}$	[24]
i_{NX}	$0.07\ gN\ g^{-1}COD_{XH,XA,XN}$	[24]

Biofilm model

The biofilm was divided into 1 μm thick layers. Each layer was assumed to have uniform distribution of biomass- and soluble components. The biomass was distributed into the biofilm layers based on measured total solids concentrations [25], qFISH and cryosection FISH images.

The concentrations of soluble components in each layer is governed by diffusion (Fick's law) and biochemical reactions. The reaction-diffusion mass balance equation for a layer in the biofilm can be written as Equation 2.

$$\frac{dS_m}{dt} = D_e \cdot \frac{(S_{m+1} - 2 \cdot S_m + S_{m-1})}{\Delta x^2} + r \qquad (2)$$

where S_m is the substrate component concentration in layer m (g m^{-3}), t is time (d), D_e is the effective diffusion coefficient of the substrate inside the biofilm (m^2 s^{-1}), Δx is the thickness of a layer (m), and r is the conversion rate of S due to biochemical reactions (g m^{-3} s^{-1}).

The effective diffusion coefficient (D_e) was calculated based on the correlation with biofilm density observed by Fan, et al. [26]

$$\frac{D_e}{D_w} = 1 - \frac{0.43 \cdot X_V^{0.92}}{11.19 + 0.27 \cdot X_V^{0.99}} \qquad (3)$$

where D_w is the diffusion coefficient in water (m^2 s^{-1}) and X_V is the biofilm density (kg TS m^{-3}).

The reaction-diffusion mass balance was solved using a finite difference method with the following boundary conditions. At the bottom of the biofilm the diffusion gradient is zero (Equation 4) and at the surface the diffusion gradient is governed by mass transfer from the bulk liquid (Equation 5).

$$\frac{dS_0}{dx} = 0 \qquad (4)$$

$$\frac{dS_L}{dx} = \frac{D_w}{\delta_{BL}} \cdot (S_B - S_L) \qquad (5)$$

where S_0 is the concentration at the bottom of the biofilm (g m^{-3}), S_L is the concentration at the outer surface of the biofilm (g m^{-3}), δ_{BL} is the liquid-granule boundary layer thickness (m), and S_B is the concentration in the bulk liquid (g m^{-3}).

Physical parameter values used as default input to the model are shown in Table S6.

Table S6. Default input values for physical parameters used in the DO model.

Coefficient	Value	Reference
D_{W_O}	$3.01 \cdot 10^{-9}$ m^2 s^{-1}	[27]
D_{W_N}	$1.62 \cdot 10^{-9}$ m^2 s^{-1}	[27]
D_{W_A}	$1.74 \cdot 10^{-9}$ m^2 s^{-1}	[27]
D_{W_C}	$1.24 \cdot 10^{-9}$ m^2 s^{-1}	[28]
f_{VS}	0.2-0.8	Different scenarios tested
fX_H	76.8% and 54.8%	Calculated ($1-fX_A-fX_N-fX_{anammox}$)
fX_A	7.4% and 22.6%	For Z400 and Z50
fX_N	12.9% and 22.6%	For Z400 and Z50
$S_{O,bulk}$	5.5 and 5.6 g m^{-3}	For Z400 and Z50 in batch tests
$S_{N,bulk}$	0.5 and 0.8 g m^{-3}	For Z400 and Z50 in batch tests
$S_{A,bulk}$	30.7 and 31.3 g m^{-3}	For Z400 and Z50 in batch tests
$S_{C,bulk}$	10 g m^{-3}	Assumed
L	379 and 45 µm	For Z400 and Z50 (based on FISH)
δ_{BL}	$(1.6\text{-}16.3) \cdot 10^{-6}$ m	Fitted values

Z50 carriers

The Z50 carriers had a biofilm density of 3.3 gTS/m^2 and an average thickness of 45 µm [25]. Cryosection FISH images of the Z50 carriers showed a stratification of the biofilm density, which was used as input to the model (Supplementary Fig. S3a). However, there was no clear stratification of the distribution of X_H, X_A and X_N. We therefore assumed that these components were distributed homogenously throughout the biofilm. The fractions X_A and X_N of the active biomass were both determined to be 22.6% by qFISH; 54.8% was assumed to be X_H.

Z400 carriers

The Z400 carriers had a biofilm density of 14.1 gTS/m^2 and an average thickness of 379 µm [25]. The fractions of X_A, X_N, and anammox of the active biomass were 7.4, 12.9, and 2.9% as determined by qFISH. The remaining part of the active biomass, i.e. 76.8%, was assumed to

be X_H. The biomass components were distributed in the biofilm based on stratification data obtained using cryosection FISH images of the biofilm (Supplementary Fig S3b and S3c).

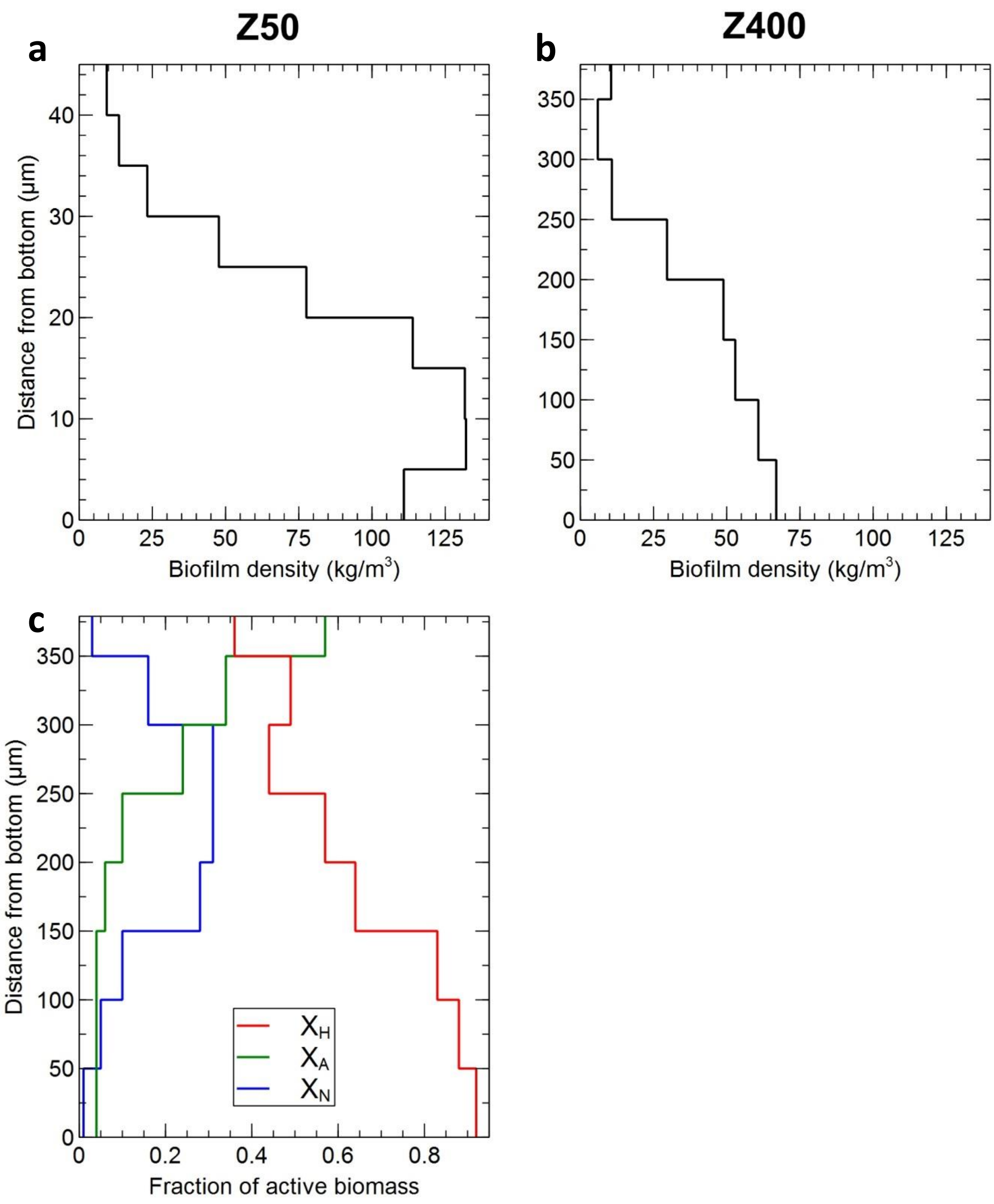

Fig. S3. Density profiles and biomass distribution for the DO model. Biofilm density profiles (total dry solids) in the Z50 (**a**) and Z400 (**b**) biofilms respectively. (**c**) Assumed biomass distribution in the Z400 biofilm based on input from qFISH and cryosection FISH images

Solving the model

The model was programmed and solved in Python 3.3 with the package Numpy 1.9 installed. Two important input parameter values were unknown. The fraction of the total dry solids that was live, active biomass (f_{VS}) and the thickness of the diffusion boundary layer between the bulk liquid and the biofilm (δ_{BL}). Using data from the nitrogen transformation activity tests as input, the model was solved for f_{VS} values ranging from 0.2 to 0.8. For each f_{VS}, the δ_{BL} that resulted in a simulated ammonium consumption rate that equaled the experimentally measured value was determined. For the Z50 biofilms, the δ_{BL} ranged from 1.6 μm to 6.8 μm for f_{VS} of 0.2 and 0.8, respectively. For the Z400 biofilms, the δ_{BL} ranged from 8.9 μm to 16.3 μm for f_{VS} of 0.2 and 0.8, respectively. The DO concentration profiles that resulted from each set of f_{VS} and δ_{BL} values are shown as shaded region in Figure 6 in the main article. These concentration profiles shows the depth to which DO can penetrate in the biofilms. The width of the shaded regions shows the uncertainty of the model estimations. The model was solved for the conditions in the nitrogen transformation activity batch tests because detailed information about ammonium oxidation rates for the two types of carriers was available from those experiments. The conditions were very similar in the pilot-scale reactor; however, the average DO concentration was slightly lower (5 mg/L in pilot, 5.5-5.6 mg/L in batch tests). This means that the DO penetration into the biofilms may have been somewhat lower in the pilot-scale reactor.

REFERENCES

1 Albertsen, M., Karst, S. M., Ziegler, A. S., Kirkegaard, R. H. & Nielsen, P. H. Back to Basics – The Influence of DNA Extraction and Primer Choice on Phylogenetic Analysis of Activated Sludge Communities. *PLoS ONE.* **10**, e0132783 (2015).

2 Větrovský, T. & Baldrian, P. The Variability of the 16S rRNA Gene in Bacterial Genomes and Its Consequences for Bacterial Community Analyses. *PLoS ONE.* **8**, e57923 (2013).

3 Baptista, J. D. C. *et al.* Agreement between amoA Gene-Specific Quantitative PCR and Fluorescence In Situ Hybridization in the Measurement of Ammonia-Oxidizing Bacteria in Activated Sludge. *Appl. Environ. Microbiol.* **80**, 5901-5910 (2014).

4 Persson, F., Suarez, M., Hermansson, M., Plaza, E. & Wilén, B.-M. Community structure of partial nitritation-anammox biofilms at decreasing substrate concentrations and low temperature *Microb Biotechnol.* **154**, 267-273 (2016).

5 Morgenroth, E. *et al.* Effect of long-term idle periods on the performance of sequencing batch reactors. *Water Sci. Technol.* **41**, 105 (2000).

6 Manz, W., Amann, R., Ludwig, W., Wagner, M. & Schleifer, K.-H. Phylogenetic Oligodeoxynucleotide Probes for the Major Subclasses of Proteobacteria: Problems and Solutions. *Syst. Appl. Microbiol.* **15**, 593-600 (1992).

7 Behnam, F., Vilcinskas, A., Wagner, M. & Stoecker, K. A Straightforward DOPE (Double Labeling of Oligonucleotide Probes)-FISH (Fluorescence In Situ Hybridization) Method for Simultaneous Multicolor Detection of Six Microbial Populations. *Appl. Environ. Microbiol.* **78**, 5138-5142 (2012).

8 Adamczyk, J. *et al.* The Isotope Array, a New Tool That Employs Substrate-Mediated Labeling of rRNA for Determination of Microbial Community Structure and Function. *Appl. Environ. Microbiol.* **69**, 6875-6887 (2003).

9 Wagner, M., Rath, G., Amann, R., Koops, H.-P. & Schleifer, K.-H. In situ Identification of Ammonia-oxidizing Bacteria. *Syst. Appl. Microbiol.* **18**, 251-264 (1995).

10 Daims, H., Nielsen, J. L., Nielsen, P. H., Schleifer, K.-H. & Wagner, M. In Situ Characterization ofNitrospira-Like Nitrite-Oxidizing Bacteria Active in Wastewater Treatment Plants. *Appl. Environ. Microbiol.* **67**, 5273-5284 (2001).

11 Lücker, S. *et al.* Nitrotoga-like bacteria are previously unrecognized key nitrite oxidizers in full-scale wastewater treatment plants. *ISME J.* **9**, 708 (2014).

12 Schmid, M., Schmitz-Esser, S., Jetten, M. & Wagner, M. 16S-23S rDNA intergenic spacer and 23S rDNA of anaerobic ammonium-oxidizing bacteria: implications for phylogeny and in situ detection. *Environ Microbiol.* **3**, 450-459 (2001).

13 Daims, H., Lücker, S. & Wagner, M. daime, a novel image analysis program for microbial ecology and biofilm research. *Environ Microbiol.* **8**, 200-213 (2006).

14 Hugerth, L. W. *et al.* DegePrime, a Program for Degenerate Primer Design for Broad-Taxonomic-Range PCR in Microbial Ecology Studies. *Appl. Environ. Microbiol.* **80**, 5116-5123 (2014).

15 Caporaso, J. G. *et al.* Global patterns of 16S rRNA diversity at a depth of millions of sequences per sample. *Proc Natl Acad Sci USA.* **108**, 4516-4522 (2011).

16 Kozich, J. J., Westcott, S. L., Baxter, N. T., Highlander, S. K. & Schloss, P. D. Development of a Dual-Index Sequencing Strategy and Curation Pipeline for Analyzing Amplicon Sequence Data on the MiSeq Illumina Sequencing Platform. *Appl. Environ. Microbiol.* **79**, 5112-5120 (2013).

17 Edgar, R. C. UNOISE2: improved error-correction for Illumina 16S and ITS amplicon sequencing. Preprint at https://www.biorxiv.org/content/early/2016/10/15/081257 (2016).

18 Edgar, R. SINTAX: a simple non-Bayesian taxonomy classifier for 16S and ITS sequences. Preprint at https://www.biorxiv.org/content/early/2016/09/09/074161 (2016).

19 Quast, C. *et al.* The SILVA ribosomal RNA gene database project: improved data processing and web-based tools. *Nucleic Acids Res.* **41**, D590-D596 (2013).

20 Gujer, W. & Henze, M. Activated Sludge Modelling and Simulation. *Water Sci. Technol.* **23**, 1011-1023 (1991).

21 Gujer, W., Henze, M., Mino, T. & Loosdrecht, M. v. Activated sludge model No. 3. *Water Sci. Technol.* **39**, 183-193 (1999).

22 Moussa, M. S., Hooijmans, C. M., Lubberding, H. J., Gijzen, H. J. & van Loosdrecht, M. C. M. Modelling nitrification, heterotrophic growth and predation in activated sludge. *Water Res.* **39**, 5080-5098 (2005).

23 de Kreuk, M. K., Picioreanu, C., Hosseini, M., Xavier, J. B. & van Loosdrecht, M. C. M. Kinetic model of a granular sludge SBR: Influences on nutrient removal. *Biotechnol. Bioeng.* **97**, 801-815 (2007).

24 Henze, M., Gujer, W., Mino, T. & van Loosdrecht, M. *Activated Sludge Models ASM1, ASM2, ASM2D, ASM3*. Vol. 5 (IWA publishing, 2000).

25 Piculell, M. *et al.* The inhibitory effects of reject water on nitrifying populations grown at different biofilm thickness. *Water Res.* **104**, 292-302 (2016).

26 Fan, L. S., Leyva-Ramos, R., Wisecarver, K. D. & Zehner, B. J. Diffusion of phenol through a biofilm grown on activated carbon particles in a draft-tube three-phase fluidized-bed bioreactor. *Biotechnol. Bioeng.* **35**, 279-286 (1990).

27 Williamson, K. & McCarty, P. L. Verification Studies of the Biofilm Model for Bacterial Substrate Utilization. *J Water Pollut Control Fed.* **48**, 281-296 (1976).

28 Perry, R. & Perry, J. *Chemical Engineers' Handbook*. 4 edn, (McGraw-Hill, 1963).
